# Perceived Stress, Behavior, and Body Mass Index Among Adults Participating in a Worksite Obesity Prevention Program, Seattle, 2005–2007

**DOI:** 10.5888/pcd9.120001

**Published:** 2012-10-04

**Authors:** Wendy E. Barrington, Rachel M. Ceballos, Sonia K. Bishop, Bonnie A. McGregor, Shirley A.A. Beresford

**Affiliations:** Author Affiliations: Rachel M. Ceballos, Bonnie A. McGregor, Shirley A.A. Beresford, University of Washington, School of Public Health and Fred Hutchinson Cancer Research Center, Seattle, Washington; Sonia K. Bishop, Fred Hutchinson Cancer Research Center, Seattle, Washington.

## Abstract

**Introduction:**

Stress in numerous contexts may affect the risk for obesity through biobehavioral processes. Acute stress has been associated with diet and physical activity in some studies; the relationship between everyday stress and such behavior is not clear. The objective of this study was to examine associations between perceived stress, dietary behavior, physical activity, eating awareness, self-efficacy, and body mass index (BMI) among healthy working adults. Secondary objectives were to explore whether eating awareness modified the relationship between perceived stress and dietary behavior and perceived stress and BMI.

**Methods:**

Promoting Activity and Changes in Eating (PACE) was a group-randomized worksite intervention to prevent weight gain in the Seattle metropolitan area from 2005 through 2007. A subset of 621 participants at 33 worksites provided complete information on perceived stress at baseline. Linear mixed models evaluated cross-sectional associations.

**Results:**

The mean (standard deviation [SD]) Perceived Stress Scale-10 score among all participants was 12.7 (6.4), and the mean (SD) BMI was 29.2 kg/m^2 ^(6.3 kg/m^2^). Higher levels of perceived stress were associated with lower levels of eating awareness, physical activity, and walking. Among participants who had low levels of eating awareness, higher levels of perceived stress were associated with fewer servings of fruit and vegetables and greater consumption of fast food meals.

**Conclusion:**

Dietary and physical activity behaviors of workers may be associated with average levels of perceived stress. Longitudinal studies are needed, however, to support inclusion of stress management or mindfulness techniques in workplace obesity prevention efforts.

## Introduction

The high prevalence of obesity is a major public health problem because of the association of obesity with chronic health conditions such as coronary heart disease, type 2 diabetes, and some cancers (1). The increase in prevalence during the last several decades more likely stems from environmental factors at multiple levels than genetic changes (2). More than one-third of the American adult population is obese (3); long-term behavior-change interventions that focus on weight reduction have had limited success in reducing overweight and obesity. From a socioecological perspective, behavioral interventions may have limited success because they inadequately consider environmental factors (4).

Research interest in the potential role of stress in health is growing. Stress is an individual-level factor associated with environmental phenomena and individual-level disease processes (5). Stress occurs when environmental demands tax or exceed the adaptive capacity of an organism; the demands result in physiologic or psychological processes that put the organism at risk for disease (5). Adults experience numerous types of stress (eg, work, finances, family), and each type contributes to overall stress. Although types of stress may vary from person to person and group to group, overall measures of stress allow generalized comparisons. Measures of stress are imperfect, but measures of perceived stress are valuable because they account for differences in the appraisal of what is stressful, exposure to stressors, and coping ability (6).

Perceived stress is associated with direct changes to both physiologic (eg, hormonal response) and psychological processes. Chronically elevated levels of perceived stress affect cortisol levels, which have been associated with increased risk for central obesity (7). Evidence for an association between stress and physical activity behaviors is mixed (8), and more testing of physical activity theory is needed to identify inconsistencies in the literature (4,9). Experimental studies have demonstrated that acute stress affects dietary behaviors, especially among people with certain eating behaviors, such as restrained eating (ie, intentional caloric restriction) (7,10). The way in which food is consumed has also been associated with overweight and obesity in epidemiologic settings (11,12). Such behaviors include emotional eating (ie, eating to manage feelings) and task eating (ie, eating while doing other activities). These behaviors connote a level of distraction — a lack of eating awareness — that has also been linked to obesity (13). A lack of eating awareness may co-occur with a hectic or stressful lifestyle and facilitate unhealthy dietary choices.

Some types of stress, such as work stress, have been associated with obesity-related behaviors among adults (14-16). Evaluating the relationship between perceived stress and obesity-related behaviors in a working population may be especially valuable because this population likely experiences numerous types of stress and a broader range of stress exposure (17,18). More than half of adults in the United States and in King County, Washington, our study population, are employed; they typically spend more than half of their waking hours at work (19). The objective of this study was to examine associations between perceived stress, dietary behavior, physical activity, self-efficacy, and body mass index (BMI) among healthy working adults. Secondary objectives were to explore whether eating awareness modified the relationship between perceived stress and dietary behavior. We hypothesized that behavioral and self-efficacy indices of dietary behavior and physical activity would be associated with overall perceived stress (Figure). 

**Figure:**
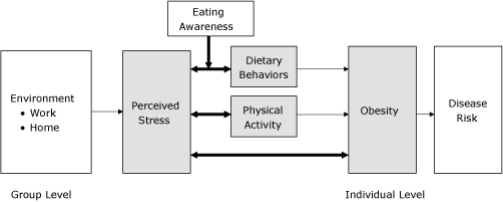
Hypothesized conceptual model of biobehavioral association of perceived stress with obesity. Bolded arrows indicate relationships assessed in this study.

## Methods

### Study participants and procedures

PACE (Promoting Activity and Changes in Eating) evaluated associations of stress and obesity-related behaviors in a population of sedentary, mostly non-Hispanic white workers in metropolitan Seattle. PACE was a large group-randomized intervention to prevent weight gain in 34 worksites employing 2,900 workers. Baseline data collection took place from 2005 through 2007; design and eligibility criteria are described elsewhere (11). All study participants completed a standard questionnaire on demographic characteristics, dietary behaviors, eating awareness, physical activity, and self-efficacy. Investigators approached a random subsample of 1,263 employees at 34 worksites to complete additional measures; 864 employees at 34 worksites agreed to participate. Of those, 33 worksites comprising 627 participants completed the 10-item Perceived Stress Scale (PSS-10); 621 participants had no missing data on PSS-10 items. We conducted our analyses among this group. The University of Washington and Fred Hutchinson Cancer Center institutional review boards reviewed and approved all study protocols and materials. All study participants provided written informed consent.

### Measures

#### Perceived stress

We assessed the level of stress perceived by each participant in the past 30 days through the PSS-10 (20). Each item (eg, “In the last month, how often have you been angered because of things that were outside of your control?”) was rated on a 5-point Likert scale that ranged from 0 (never) to 4 (very often). Possible total scores range from 0 to 40; higher scores indicate higher levels of perceived stress. Studies of human social stress and health outcomes use the PSS-10, which has good reliability (α ranges from 0.84 to 0.86) (20).

#### Body mass index

We calculated BMI (kg/m^2^) by using heights and weights measured by investigators at worksites during a proctored survey or other assessment or at a height and weight clinic.

#### Dietary behavior

We used indices of dietary behavior instead of actual consumption measurements as our main outcome measures. The average number of fruit and vegetable servings consumed per day by participants was assessed by using the National Cancer Institute 5 A Day 7-question fruit and vegetable assessment tool (21) and a single-item summary question (11). Fast food meals and soft-drinks consumed were also measured via single-item (22,23).

#### Eating awareness

Eating awareness was assessed by measuring the frequency of task eating. We assessed the frequency of task eating by using a single question: “How often do you eat food (meals or snacks) while doing another activity, for example, watching TV, working at a computer, reading, driving, playing video games?” Respondents chose from a 5-point Likert scale ranging from 1 (never) to 5 (always). We then defined a low level of eating awareness as task eating always or most of the time and a high level of eating awareness as task eating sometimes, seldom, or never. Eating awareness may be a correlate of obesity (11,12), but validation and reliability studies are lacking.

#### Physical activity 

We used 3 measures of physical activity. Two measures were from the Godin Leisure-Time Exercise Questionnaire (24). We asked respondents to indicate the number of times in the past 7 days during their free time they engaged for at least 10 minutes in exercise categorized as strenuous (eg, running, jogging), moderate (eg, fast walking, baseball), and mild (eg, yoga, easy walking). We computed a physical activity score according to the recommended protocol, which uses metabolic equivalent task (MET) units; higher scores indicate more activity (24). Scores ranged from 0 to more than 180, but we capped the maximum at 180 to minimize the effect of outliers. We also asked respondents to indicate the frequency of sweat-inducing activity in the last 7 days as “often,” “sometimes,” or “never/rarely.” We defined regular engagement in sweat-inducing exercise as behavior that occurred often. The third measure of physical activity was frequency of walking. We used a single-item, adapted from the International Physical Activity Questionnaire (IPAQ): “During the last 7 days, on how many days did you walk for at least 10 minutes at a time?” These 3 physical activity measures have been previously validated (25,26).

#### Self-efficacy for behavior change

Self-efficacy, a determinant and consequence of behavior change (9), is integral to fruit and vegetable consumption (27), the ability to make healthier choices about weight (28), and the uptake and maintenance of physical activity behaviors (29) in adults. We assessed self-efficacy to change dietary and physical activity behavior by using 2 items: “How sure are you that you can stick to a plan to monitor your eating choices on a regular basis?” and “How sure are you that you can increase your level of physical activity on a regular basis?” Responses were on a 5-point Likert scale ranging from “not sure” to “extremely sure.”

### Statistical analyses

We conducted linear mixed-model analyses to examine the hypothesized associations. In all models, we calculated predicted means and 95% confidence intervals, adjusting for age, sex, race, and education (as fixed effects) and a worksite random effect. We additionally included an interaction term between perceived stress and eating awareness in each dietary behavior model to explore whether eating awareness modified the relationship between perceived stress and dietary behavior. Analyses were then stratified by low and high levels of eating awareness. When modeling total walking behavior, we also included manual occupation as a fixed effect because this behavior was not evenly distributed among occupational groups. Variables for BMI and the number of fast food meals and soft drinks consumed per week were log-transformed to avoid heteroscedasticity of residual errors. Variables were then back-transformed. Each predicted mean difference was related to a 9-point increase in the PSS-10 score; the 9 points correspond to the interquartile range (approximately 8.0 to 17.0). We used the Wald test to determine whether predicted mean differences or multiplicative interaction terms were significantly different from zero. We used SAS version 9.2 (SAS Institute Inc*,* Cary, North Carolina*) *for all analyses.

## Results

Among PACE participants, 82.7% were non-Hispanic white; almost half were college graduates and had a household income of $75,000 or more (Table 1). The mean (SD) PSS-10 score among all participants was 12.7 (6.4), and the mean (SD) BMI was 29.2 (6.3). A higher level of stress was associated with a higher percentage of participants who engaged in task eating always or most of the time, a lower Godin leisure-time exercise score, a lower percentage of participants who regularly engaged in sweat-inducing exercise, and fewer minutes walked per week (Table 2). We found a significant interaction between perceived stress and eating awareness in relation to the 5 A Day (*z* = −3.01, *P* = .003), single-item summary of fruit and vegetable consumption (*z* = −2.77, *P* = .006), and fast food consumption (*z* = 3.00, *P* = .003). Among participants who had a low level of eating awareness, higher levels of perceived stress were associated with fewer servings of fruit and vegetables per day and greater consumption of fast food meals (Table 3). We also found a significant interaction between perceived stress and eating awareness in relation to soft drink consumption (*z* = 2.97, *P* = .003); associations between perceived stress and soft drink intake in stratified analyses, however, were not significant.

**Table 1:** Baseline Demographic and Health-Related Characteristics of a Subset of Participants in Promoting Activity and Changes in Eating (PACE), Seattle, 2005–2007^a^

Characteristic	Total (n = 621)	Men (n = 264)	Women (n = 357)
**Demographic**			
**Age, y**			
18–34	141 (22.8)	48 (18.1)	93 (26.3)
35–44	158 (25.5)	66 (24.9)	92 (25.9)
45–54	200 (32.2)	98 (37.0)	102 (28.7)
55–65	121 (19.5)	53 (20.0)	68 (19.1)
**Race/ethnicity**			
White	505 (82.7)	222 (85.5)	283 (80.6)
African American	35 (5.7)	9 (3.4)	26 (7.4)
Hispanic/Latino	25 (4.6)	9 (3.4)	16 (4.5)
Asian	27 (4.5)	9 (3.4)	18 (5.2)
Other	19 (3.1)	11 (4.2)	8 (2.3)
**Education**			
<High school, high school graduate, or GED	70 (11.3)	31 (11.7)	39 (11.0)
Some college or technical college	250 (40.3)	94 (35.5)	156 (43.8)
College graduate	213 (34.3)	92 (34.7)	121 (34.0)
Postgraduate or professional degree	87 (14.0)	47 (17.7)	40(11.2)
**Household income, $**			
<50,000	149 (27.2)	44 (18.8)	105 (31.9)
50,000–74,999	131 (23.6)	57 (26.2)	74 (22.1)
75,000–100,000	105 (18.9)	43 (18.9)	62 (19.2)
>100,000	166 (30.3)	81 (36.0)	85 (26.8)
**Manual occupation^b^**	79 (13.6)	58 (20.9)	21 (5.8)
**Health-Related**			
**Perceived Stress Score-10,^c^ mean (SD)**	12.7 (6.4)	11.6 (6.0)	13.6 (6.4)
**Body mass index,^d^ mean (SD), kg/m^2^**	29.2 (6.3)	28.7 (4.2)	29.2 (6.7)
**Dietary behaviors**			
No. of fruit and vegetable servings per day, mean (SD)			
5 A Day	3.2 (3.1)	3.2 (1.9)	3.4 (2.1)
Single item summary	3.1 (1.7)	2.9 (1.6)	3.3 (1.6)
No. of fast food meals consumed per week, mean (SD)	0.5 (0.6)	0.6 (0.7)	0.5 (0.4)
No. of soft drinks consumed per week, mean (SD)	3.7 (4.4)	3.6 (4.0)	3.9 (4.4)
**Eat while doing another activity all or most of the time**	127 (20.3)	69 (26.1)	58 (15.5)
**Physical activity**			
Godin leisure-time exercise score,^e^ mean, (SD)	28.7 (22.4)	33.8 (21.0)	26.2 (20.4)
Regularly engage in free-time sweat-inducing exercise	155 (24.7)	81 (33.8)	74 (21.2)
No. of walking minutes per week, mean (SD)	515.2 (502.2)	547.9 (487.3)	495.8 (490.8)
**Self-efficacy**			
Very or extremely sure they can monitor eating choices	364 (9.0)	34 (11.5)	20 (6.3)
Very or extremely sure they can increase physical activity	57 (9.5)	31 (12.4)	26 (7.0)

Abbreviations: GED, General Educational Development; SD, standard deviation.

^a^ Employees at 33 worksites completed Perceived Stress Scale-10. Characteristics were averaged within and among worksites for those that provided data from Perceived Stress Scale-10; totals may not add to total sample because of missing data. Values are n (%) unless otherwise indicated.

^b^ Includes machine operators, mechanics/technicians, building trade, construction and labor, and service workers.

^c^ Perceived Stress Score-10 ranges from 0 to 40; 40 represents the greatest level of perceived stress.

^d^ Body mass index calculated from measured height and weight.

^e^ Godin Leisure-Time Exercise Questionnaire (24) was scored by number of times in past week spent in strenuous, moderate, or mild exercise for at least 10 minutes. Score ranges from 0 to 180 metabolic equivalent task (MET) units; 180 represents the greatest level of physical activity.

**Table 2:** Predicted Mean Differences in Health-Related Characteristics According to Difference in Perceived Stress,^a^ Promoting Activity and Changes in Eating (PACE), Seattle, 2005–2007

Characteristic	Mean Difference (95% CI)	*P* Value^b^
**Body mass index^c^, kg/m^2^**	0.31 (−0.31 to 0.97)	.33
**Dietary behavior**		
No. of fruit and vegetable servings per day		
5 A Day	−0.19 (−0.41 to 0.04)	.11
Single-item summary	−0.12 (−0.27 to 0.04)	.11
No. of fast food meals consumed per week	0.02 (−0.02 to 0.07)	.34
No. of soft drinks consumed per week, n	0.14 (−0.10 to 0.50)	.32
**Eat while doing other activities all or most of the time, percentage point**	7.1 (1.4 to 13.1)	.01
**Physical activity**		
Godin leisure-time exercise score score^d^	−3.1 (−5.7 to −0.4)	.02
Regularly engage in free-time sweat-inducing exercise, percentage point	−5.3 (−7.9 to −0.01)	.02
No. of walking minutes per week^e^	−49.8 (−80.7 to −7.7)	.02
**Self-efficacy**		
Very or extremely sure they can monitor eating choices, percentage point	−0.04 (−10.5 to 0.01)	.14
Very or extremely sure they can increase physical activity, percentage point	−0.05 (−0.12 to 0.001)	.06

Abbreviation: CI, confidence interval.

^a^ Predicted mean differences were calculated by linear mixed models using a link function adjusted for individual age, sex, race, and education and a worksite random effect. Each mean value corresponds to a 9-point increase in the Perceived Stress Score-10 (PSS-10). For example, for each 9-point increase in the PSS-10, the percentage of study participants who were categorized as task eaters (eat while doing other activities all or most of the time) increased by 7.1 percentage points.

^b^ Determined by the Wald test.

^c^ Body mass index calculated from measured height and weight.

^d^ Godin Leisure-Time Exercise Questionnaire (24) was scored by measuring number of times in past week spent in strenuous, moderate, or mild exercise for at least 10 minutes. Score ranges from 0 to 180 metabolic equivalent task (MET) units; 180 represents the greatest level of physical activity.

^e^ Adjusted for manual occupation.

**Table 3:** Predicted Mean Difference in Dietary Behaviors According to Difference in Perceived Stress,^a^ by Level of Eating Awareness, Promoting Activity and Changes in Eating (PACE), Seattle, 2005–2007

	Low Level of Eating Awareness^b^		High Level of Eating Awareness^b^	
	Mean Difference (95% CI)	*P* Value^c^	Mean Difference (95% CI)	*P* Value^c^
**No. of fruit and vegetable servings per day**				
5 A Day^d^	−0.54 (−0.93 to −0.15)	.007	0.10 (−0.25 to 0.45)	.59
Single item summary^e^	−0.44 (−0.70 to −0.18)	.001	0.15 (−0.09 to 0.38)	.23
**No. of fast food meals consumed per week^f^**	0.13 (0.05 to 0.22)	.001	−0.05 (−0.10 to 0.01)	.07
**No. of soft drinks consumed per week^g^**	0.41 (−0.09 to 1.41)	.14	−0.04 (−0.25 to 0.32)	.79

Abbreviation: CI, confidence interval.

^a^ Predicted mean differences were calculated by linear mixed models using a link function adjusted for individual age, sex, race, and education and a worksite random effect. Each mean value corresponds to a 9-point increase in the Perceived Stress Score-10 (PSS-10).

^b^ Low level of eating awareness defined as task eating (eating while doing other activities such as reading or watching television) always or most of the time and a high level of eating awareness as task eating sometimes, seldom, or never.

^c^ *P* values determined by the Wald test.

^d^ *P* value for interaction = .003.

^e^ *P* value for interaction = .006.

^f^ *P* value for interaction = .003.

^g^ *P* value for interaction = .003.

## Discussion

Our sample of working adults was similar to US adults in terms of BMI (1) and perceived stress (30). Compared with the US population, however, our sample had a higher level of income and education and a higher proportion of non-Hispanic whites (11). BMI in our sample was not associated with perceived stress. The distribution of perceived stress scores, however, indicated average levels of stress (ie, levels experienced in everyday living) and does not reflect a highly stressed population (eg, shift-workers, racial/ethnic minority groups) where we would expect to find an association (31). In other words, the range of perceived stress scores in our sample setting was likely too low to detect an association. However, this same range was sufficient to detect associations with more proximal obesity-related behavioral indices.

Higher frequency of task eating was associated with higher levels of perceived stress, but overall dietary behavior and perceived stress was not associated. We also did not detect a sex interaction in the relationship between perceived stress and dietary behavior and physical activity. The relationship between perceived stress and dietary behavior may be modified by several factors, including overweight or obesity, sex, and domains of eating behavior (eg, restrained or emotional eating) (32). Women and overweight or obese people may be more likely to eat in ways in which dietary choice is influenced by stress. In controlled laboratory studies, participants, particularly women and restrained eaters, have responded to high levels of stress by consuming foods high in calories, sugar, and fat (ie, more palatable foods), fewer main meals, and fewer portions of vegetables (7,10,33,34).

Growing evidence suggests that stress’s influence on *what* you eat depends on *how* you eat. People characterized as stress- or emotional-eaters tend to choose calorically dense foods to blunt their stress response or reduce negative emotions (7). The relationship between restrained eating and stress is more complex; retrained eaters tend to eat less during normal conditions and overeat when stressed (7). We propose that variables used to operationalize these eating behaviors share the underlying construct of lack of eating awareness. That is, when energy-dense eating serves as reward during times of stress or negative emotions, assessment and moderation (ie, awareness) of caloric intake is absent. The interaction of low eating awareness on the association between stress and dietary behaviors in our data supports this line of thinking.

Higher levels of perceived stress were also associated with lower levels of physical activity. These findings are consistent with those found in most previous studies (8), although the direction of the relationship has not been determined. Stress may inhibit people from engaging in physical activity, or lack of physical activity may lead to increased stress levels, or both of these scenarios may occur. Numerous cross-sectional studies document both a presence and lack of an association between higher levels of stress and lower levels of physical activity (8). Of the null studies, however, two (35,36) measured perceived stress among older persons, who tend to have lower levels of stress (30), which decreased the likelihood of detecting an association. A pos thoc analysis (37) found that stress was more strongly associated with physical activity among people aged 30 to 44 (a group hypothesized as having higher levels of stress) than among people aged 18 to 29 or 45 or older. Higher levels of stress also predicted lower levels of physical activity among a prospective cohort of people at risk for diabetes (38).

Higher levels of stress have been associated with a lack of adherence to physical activity (35). Among a study of postmenopausal women, stress was modestly associated with failure to return to activity among those who had stopped (35). Studies on middle-aged women (39) and university students (39) found a decrease in exercise duration and an increase in the number of missed exercise opportunities as stress increased. Self-efficacy for dietary behavior and physical activity has also been negatively associated (but not significantly) with perceived stress (8,38). These findings support the idea that stress inhibits positive health behaviors, ultimately affecting one’s risk for obesity; they are also consistent with studies on stress and obesogenic behaviors among adults. Lower levels of social support among workers were associated with lower fruit and vegetable intake and lower levels of leisure-time physical activity (14), whereas greater work demands were associated with lower levels of physical activity (16), higher levels of task eating (women only) (15), and higher levels of fast food consumption (men only) (15) among working adults.

This study has several limitations, including its secondary cross-sectional design and the potential issue of multiple comparisons. Confirmatory analyses are needed, especially within a longitudinal setting. We did not include other measures of stress-related eating behaviors (eg, emotional eating) because evaluating the relationship between stress and eating was not the primary objective of the PACE intervention. Alcohol consumption, a potentially confounding variable in the relationship between stress and BMI, was also not measured (22). Using single items may also have been a disadvantage and perhaps relevant to our measures of self-efficacy. It may be important, for example, to measure both confidence in and barriers to self-efficacy because each may relate to different aspects of adopting behaviors in this population (4,9). Finally, most of our data were based on self-report. By using behavioral indices, we sought to minimize the biases potentially introduced by self-report and decrease the burden on the participant. Strengths of the study included adjustment for the multilevel data, analyses accounting for variance at worksite and individual levels, and use of physical measurements for BMI calculation.

In this study, perceived stress was associated with several obesogenic behaviors among mostly non-Hispanic white working adults with average levels of everyday stress. Our data provide insight into the potential role of even average levels of everyday stress in dietary, eating, and physical activity behaviors. The association of perceived stress and these behaviors is likely greater in people who have higher levels of stress. Further exploration of the role of stress in occupational groups in which excessive obesity-related behaviors have been documented may benefit future intervention strategies. Our findings may also suggest that including stress management and/or mindfulness techniques in worksite behavior-change interventions could improve program effectiveness.
